# *Staphylococcus aureus* carriage is associated with microbiome composition in the nares and oropharynx, not the hand, of monozygotic twins

**DOI:** 10.3389/frmbi.2024.1457940

**Published:** 2025-01-20

**Authors:** Mark R. Dalman, W. Brian Simison, Danny Nielsen, Sabana Bhatta, Noor Ramahi, Clair Yee, Dipendra Thapaliya, Jhalka Kadariya, Shanice Cheatham, Hailee Olson, Tara C. Smith

**Affiliations:** ^1^ College of Podiatric Medicine, Kent State University, Kent, OH, United States; ^2^ Center for Comparative Genomics, California Academy of Sciences, San Francisco, CA, United States; ^3^ Department of Natural Resources and Environmental Science, University of Nevada, Reno, Reno, NV, United States; ^4^ College of Public Health, Kent State University, Kent, OH, United States

**Keywords:** microbiome, 16S rRNA, *Staphylococcus aureus*, monozygotic twins, nares, oropharynx, hand, carriage

## Abstract

**Background:**

*Staphylococcus aureus* is a gram-positive bacterium commonly found in the nares and oropharynx of one in three individuals and has the potential to cause significant health problems. With antibiotic-resistant strains causing 11,000 deaths yearly and ~2% of the population nasally colonized with methicillin-resistant *S. aureus*, a search for predictive markers and associative relationships between carriage have been long-sought goals. Within our study, we leveraged monozygotic twin participants in concert with multi-site microbiome analyses to characterize the impacts of *S. aureus* on composition.

**Results:**

We recruited 147 monozygotic twin pairs and characterized three sites, i.e., the nares, oropharynx, and hand microbiomes, using 16S rRNA v3-v4 sequencing in addition to *S. aureus* carriage status. The prevalence of *S. aureus* was highest in the oropharynx followed by nares and hand with concordance between twin pairs highest in the nares, followed by oropharynx. The detection of *S. aureus* was statistically correlated with differences in microbiome composition across sites, as indicated by beta diversity and DESeq2 analyses. Microbiome composition was most similar in twins’ nares that were *S. aureus* culture-positive concordant, whereas twins that were culture-negative concordant had the most similarity in the oropharynx. Of significance, *Moraxella nonliquefacians* and *Capnocytophaga* were inversely associated with *S. aureus* in the nares and oropharynx, respectively.

**Conclusions:**

This improved understanding of *S. aureus* colonization in nares, oropharynx, and hand microbiomes in monozygotic twin pairs is a further step towards unraveling the degree to which the microbiome is influenced by host genetics and *S. aureus* carriage.

## Introduction

Symbiotic relationships between Eukaryotes and Prokaryotes have a long history (Baldo and Werren, 2021). Recent research has deepened our understanding of microbial colonization dynamics in relation to human health and disease (Campbell and Koch, 2017). The human microbiome, a community of over 10^14^ microbial cells (Sender et al., 2016), is passed from generation to generation (Rosenberg and Zilber-Rosenberg, 2016). It plays a critical role in priming the immune system (Round et al., 2010) and even shaping metabolism (Dabke et al., 2019). To better understand perturbations from normal healthy microbiomes (Kong and Segre, 2017; Oh et al., 2016), in 2008, the Human Microbiome Project (HMP1) sought to establish microbial compositional characteristics across core body sites (i.e., nasal, oropharyngeal, and skin). In doing so, studies have noted that certain microbiome perturbations, such as inverse relationships involving *Staphylococcus epidermidis* interfering with colonization of microbes such as *Leishmania major* (Naik et al., 2012) and methicillin-resistant *Staphylococcus aureus* (MRSA) (Johnson et al., 2015; Park et al., 2011) can impact composition.

*S. aureus* is a major nosocomial pathogen, contaminating surfaces (Dalman et al., 2019) and is even asymptomatically carried by over a third of the population (Vanegas et al., 2021). In the U.S., it causes over 11,000 deaths and 80,000 invasive infections annually (Dantes et al., 2013), commonly colonizing the nares and oropharynx of individuals and posing a risk for active infections. *S. aureus* is known to inhibit colonization by other bacterial species through antimicrobial peptides and toxins, potentially causing dysbiosis (Garrett and Palmer, 2024; Park et al., 2011). Our group has identified reservoirs of methicillin-sensitive and resistant strains (MSSA and MRSA) in human and environmental samples (Kates et al., 2019; Thapaliya et al., 2017); however, the associated impact on composition remains underexplored. Twin studies offer a controlled framework to explore these associations, providing insights into how host genetics influence microbial communities and *S. aureus* carriage.

Twin studies provide a valuable framework for investigating the relative contributions of genetic and environmental factors to complex traits, such as disease susceptibility and physiological characteristics (Galton, 1876; Ganna et al., 2013). By eliminating genetic variability, monozygotic (MZ) twins are particularly well-suited for studying microbiome composition and exploring associations with *S. aureus* carriage. Studies focused exclusively on monozygotic twins have advanced our understanding of the oral microbiota, revealing minimal differences in *S. aureus* presence in saliva (Bockmann et al., 2011; Wu et al., 2018). In contrast, traditional twin studies incorporating dizygotic (DZ) twins have shown mixed results, with some studies suggesting a modest genetic influence on *S. aureus* nasal carriage (Andersen et al., 2012), while other studies in closed genetic pools in Amish communities suggest that environmental factors play a larger role (Roghmann et al., 2011). Additionally, genetic studies have further identified variants associated with *S. aureus* susceptibility, such as single nucleotide variants (SNVs) in the *KAT2B* (linked to intermittent carriage), *PDE4B*, *VRK1*, and *BCL11B* genes (Brown et al., 2015; Ye et al., 2014), suggesting that genetic factors may be inherited and influence both *S. aureus* carriage and microbial composition.

The aim of this study was to characterize the microbial community of the nares, oropharynx, and hands in monozygotic twins, and to quantify any potential effect of *S. aureus* colonization on microbiome composition. We predicted that microbiome composition and complexity would differ in *S. aureus* culture-positive participants, potentially due to competitive interactions. Additionally, we predicted that twin pairs would show similar microbiome compositions in the nares and oropharynx, irrespective of *S. aureus* carriage status. This observational study, rather than testing causative mechanisms such as competitive exclusion, provides a controlled framework to assess associations between *S. aureus* presence and microbiome characteristics across multiple different body sites in only monozygotic twins. By focusing on genetically identical pairs, we minimize confounding genetic variability to better explore microbial composition and its host associations.

## Materials and methods

### Ethical statement, sample collection, and processing

All study protocols were approved by the Kent State University Institutional Review Board (#16-293). We recruited 147 monozygotic twin pairs (n=294) at the Twins Day Festival in Twinsburg, OH, in August 2016. Eligibility criteria included: 8 to 80 years of age, English-speaking, no antibiotic or inhaled corticosteroid use within 90 days, no nasal influenza vaccine in the past month, no active hand or upper respiratory infections, and no hospitalization exceeding 24 hours within 90 days. Participants provided written consent or assent (for minors), along with a parent’s consent.

Participants completed questionnaires on medical history, housing, and hygiene practices (**Supplementary Data Sheet 1**). They were asked to refrain from eating, drinking, chewing gum, brushing teeth (30 minutes), or washing hands (1 hour) prior to sample collection. Anterior nares and oropharynx swabs were collected in duplicate, along with dominant hand samples using the glove juice technique (Leyden et al., 1989; Kampf et al., 2013). All the samples were processed at the Kent State University (KSU) Smith Emerging Infections Laboratory on the day of their collection. Anterior nares and oropharynx swabs were collected with sterile nylon flocked swabs (Copan Diagnostics, Murrieta, CA), and hand microbes were collected using sterile gloves and sterile peptone. All samples were subjected to a bifurcated protocol in the lab. Of the duplicate swabs taken from the anterior nares and oropharynx, one swab was subjected to bacterial DNA isolation for 16S rRNA V3-V4 sequencing, and the other duplicate swab was subjected to a culture-based technique for *S. aureus* isolation. *S. aureus* was phenotypically assessed and confirmed using a combination of agglutination and biochemical assays as recently described by Dalman et al. (2019).

### Microbiome extraction

Microbial DNA from anterior nares, oropharynx, and hand samples was isolated using the MO BIO PowerSoil DNA isolation kit (Mo BIO Laboratories Inc, Carlsbad, CA). For swabs, the swab head was vortexed in DNA extraction buffer for 10 minutes, followed by centrifugation and resuspension before proceeding with the kit protocol. DNA was quantified using the Qubit BR dsDNA kit (Invitrogen, Waltham, MA, USA) and sent to Case Western Reserve Genomics Core for 16S rRNA V3-V4 amplification, library preparation, and sequencing using 2x250 paired-end reads (Illumina MiSeq protocol). Data was uploaded to the Illumina Basespace cloud for bioinformatics analysis following the standard Illumina MiSeq protocol. Data was uploaded to the Illumina Basespace cloud server and retrieved for bioinformatics.

### Microbiome analyses and comparisons

Raw sequence reads were processed and analyzed with QIIME2 v2021.8 (Bolyen et al., 2019) using modified scripts from Amplicon SOP v2 of Microbiome Helper (Comeau et al., 2017). Raw read quality was evaluated with FASTQC v0.11.8 (Andrews, 2010) and MultiQC v1.12 (Ewels et al., 2016). Raw FASTQ reads were imported as QIIME2 artifacts (QZA format) using the QIIME2 import tool. Primers were trimmed with Cutadapt v3.4 (Martin, 2011). The reads were denoised after joining with VSEARCH v2.13.3 (Rognes et al., 2016). All sequences were trimmed to the same length prior to using Deblur v1.1.0 (Amir et al., 2017) to correct reads and identify amplicon sequence variants (ASVs). We used the Naive-Bayes approach in the scikit-learn v1.1 Python library and the SILVA database (silva-138-99-nb-classifier.qza) to assign the ASVs to taxonomy. We removed rare ASVs that had frequencies of less than 0.1% of the mean sample depth using the QIIME2 filter-features tool. This was followed by filtering out contaminants, including chloroplast, mitochondria, unclassified ASVs, and low-coverage samples using the QIIME2 filter-table tool. To confirm that the sequence depths were sufficient, we generated rarefaction curves to identify appropriate depth cutoffs with the QIIME2 alpha-rarefaction tool. Bar charts of the taxonomic abundance across samples were generated using the QIIME2 barplot tool. Differential abundances between groups were identified using ANCOM 2.0 (Mandal et al., 2015). Finally, we generated and exported a BIOM file with taxonomy as metadata using a Microbiome helper script using sed and the QIIME2 export tool. All statistical analyses were conducted in R version 4.2.0 (R Core Team, 2022).

The QIIME2 artifacts including the feature count table, phylogenetic tree, taxonomy table, and sample metadata were converted to a phyloseq (McMurdie and Holmes, 2013) object in R using the *qza_to_phyloseq* function of the qiime2R package (Bisanz, 2018). Using the *rarefy_even_depth* function of phyloseq we rarefied microbial reads to the smallest sequencing depth of all samples. Using the *plot_richness* function of phyloseq, we compared the Shannon, Chao1, and Simpson diversity estimates, and used Wilcox tests to determine significant differences. To detect differences between tissue locations, we compared alpha diversity across the hand, nares, and oropharynx samples and between *S. aureus*-positive and -negative samples. To determine if *S. aureus* carriage influenced microbial diversity within sample locations, we also compared diversity between *S. aureus* positive and negative samples and correspondingly across tissue locations.

We visualized the relative read abundances (RRA) of microbial lineages at the level of phylum, class, order, family, genus, and species across location and *S. aureus* carriage status. We first agglomerated ASV reads to the desired taxonomic level using the *tax_glom* function of phyloseq before merging sample types using the *merge_samples* function. We then transformed the read count results to RRA using the *transform_sample_counts* function of phyloseq. We then plotted RRA using ggplot (Wickham, 2016).

Monozygotic twin’s compositional differences were tested using a G-test with Yates’ continuity correction with applied Fishers exact test when contingency table <20 (Agresti, 1992; Rivals et al., 2007; Yates, 1934). Benjamini–Hochberg FDR multiple testing correction was used to control for false discovery rate along with the Newcombe–Wilson method to test confidence intervals (Newcombe, 1998). A scatter plot and corresponding R^2^ was calculated in STAMP (Parks et al., 2014; Parks and Beiko, 2010) for all monozygotic twin pairs based on their culture-dependent status [concordant (CC), discordant (DC), and not colonized] with corresponding R^2^ values cumulatively plotted for each twin pair based on site location. An R^2^ of 1.00 indicates the identical composition of features at the hierarchical level of comparison (all analyses were done at the genus level).

We used the *ordinate* function to perform Principal Coordinates Analysis (PCoA) using the Bray–Curtis method, and both weighted and unweighted UniFrac distances. We then plotted the PCoAs using the *plot_ordination* function of phyloseq. We visualized PCoAs with combinations of the first three PCoA axes. To quantify the amount of variation in microbiome composition explained by location and *S. aureus* carriage, we performed permutation analysis of variance (PERMANOVA) using 999 permutations with the *adonis2* function of the vegan package (Oksanen et al., 2020). We performed PERMANOVA using the Bray–Curtis method, and both weighted and unweighted UniFrac distance matrices.

### Differentially abundant microbiota

To detect differentially abundant microbial lineages, we used the DESeq2 package (Love et al., 2014). We performed the analysis in multiple pairwise contrasts: 1) between the nares and oropharynx in *S. aureus-*positive samples; 2) between the nares and oropharynx in *S. aureus-*negative samples; 3) between *S. aureus*-negative and -positive samples in only the nares; and 4) between *S. aureus-*negative and -positive samples in only the oropharynx. Within the *DESeq* function, we used the *Wald* test, *parametric* fitType, and *poscounts* sfType parameters. Microbial ASVs were considered significantly differentially abundant with adjusted *P* values < 0.05 (corrected using the Benjamini–Hochberg method). Differentially abundant microbial taxa were plotted for each of the above-described analyses using ggplot2 (Wickham, 2016).

### Data access

Sequences for the project can be found under BioProject ID: PRJNA1026518. Code for analysis can be found at: https://github.com/calacademy-research/Dalman_Twin_Microbiome_Scripts.

## Results

### Participant demographics and carriage rates

Full demographic data can be found in **Table 1**. A total of 294 twins (147 twin pairs) were convenience sampled at an International Twins Festival in Twinsburg, Ohio. The average cohort age was 32.5 years (95% CI: 30.4-34.6), with *S. aureus*-positive participants averaging 30.7 years (95% CI: 27.8-33.6) and culture-negative participants 34.1 years (95% CI: 31.1-37.0). A total of 220 monozygotic twins were female (74.8%) and overall, 87.8% of participants identified as white, 158 twins (54%) lived with their twin sibling, and 53% of these cohabiting twins (84/158) shared a room.

**Table 1:** Sociodemographic characteristics of monozygotic twin participants by *Staphylococcus aureus* culture-dependent status.

Sociodemographic	Overall	*S. aureus-*positive	*S. aureus-n*egative
	n, mean or proportion (95% Cl)	n, mean or proportion (95% Cl)	n, mean or proportion (95% Cl)
Age (years)	292, 32.49 (30.42- 34.56)	135, 30.66 (27.77- 33.55)	157, 34.06 (31.11- 37.02)
Family size	290, 3.51 (3.51- 3.71)	134, 3.83 (3.52- 4.14)	156, 3.24 (2.99- 3.49)
Sex			
Female	220, 74.83 (69.84- 79.82)	93, 68.38 (60.47- 76.30)	127, 80.38 (74.12- 86.64)
Male	74, 25.17 (20.18- 30.16)	43, 31.62 (23.70- 39.53)	31, 19.62 (13.36- 25.88)
Ethnicity			
Black	22, 7.48 (4.46- 10.51)	10, 7.35 (2.91- 11.80)	12, 7.59 (3.42- 11.77)
White	258, 87.76 (83.99- 91.52)	119, 2.85 (81.87- 93.13)	139, 87.97 (82.85- 93.10)
American Indian	2, 1.45 (0.36-2.53)	1, 0.73 (0.00-2.17)	1, 0.73 (0.00-2.17)
Asian	6, 4.11 (0.85-7.37)	4, 2.91 (0.08-5.74)	2, 1.45 (0.00-3.43)
Multiracial	6, 4.11 (0.85-7.37)	2, 1.45 (0.00-3.43)	4, 2.91 (0.08-5.74)
Education			
Elementary/Junior High School	45, 15.31 (11.17- 19.45)	22, 16.18 (9.91- 22.44)	23, 14.56 (9.00- 20.12)
Some High School/High School Graduate	60, 20.41 (15.77- 25.04)	29, 21.32 (14.35- 28.30)	31, 19.62 (13.36- 25.88)
Some College	58, 19.73 (15.15- 24.30)	28, 20.59 (13.71- 27.47)	30, 19.00 (12.80- 25.17)
College Graduate	87, 29.59 (24.34- 34.84)	40, 29.41 (21.66- 37.17)	47, 29.75 (22.54- 36.95)
Postgraduate-Professional	44, 14.97 (10.86- 19.70)	17, 12.5 (6.87- 18.13)	27, 17.10 (11.16- 23.02)
Sanitizer usage			
Little to No Usage	148, 52.86 (46.97- 58.74)	76, 58.91 (50.31- 67.52)	72, 47.68 (39.62- 55.74)
Daily usage	132, 47.14 (41.26- 53.03)	53, 41.10 (32.48- 49.69)	79, 52.32 (44.26- 60.38)
Current smoker			
Yes	13, 4.42 (2.06- 6.79)	5, 3.68 (0.47- 6.88)	8, 5.06 (1.61- 8.52)
No	223, 75.85 (70.93- 80.77)	100, 73.52 (66.02- 81.04)	123, 77.85 (71.30- 84.39)
Pets			
Yes	194, 66.67 (61.22- 72.11)	88, 65.19 (57.05- 73.32)	106, 67.95 (60.54- 75.35)
No	97, 33.33 (27.89- 38.78)	47, 34.81 (26.68- 42.95)	50, 32.05 (24.65- 39.46)
Immune status			
Low	83, 28.23 (23.06- 33.41)	35, 25.74 (18.29- 33.18)	48, 30.38 (23.13- 37.63)
High	211, 71.77 (66.59- 76.94)	101, 74.26 (66.82- 81.71)	110, 69.62 (62.37- 76.87)
Microbe exposure			
Low	103, 35.03 (29.55- 40.52)	44, 32.35 (24.39- 40.31)	59, 37.34 (29.27- 44.97)
High	191, 64.97 (59.48- 70.45)	92, 67.65 (59.68- 75.61)	99, 62.66 (55.03- 70.28)
Diabetic			
Yes	11, 3.81 (1.59- 6.03)	3, 2.26 (0.00- 4.80)	8, 5.23 (1.63- 8.63)
No	278, 96.19 (93.97- 98.41)	130, 97.74 (95.19- 100.00)	148, 94.87 (91.37- 98.37)
Kidney disease			
Yes	1, 0.35 (0.0- 1.03)	0, 0.00 (0.00-0.00)	1, 0.64 (0.00- 1.90)
No	288, 99.65 (98.97- 100.00)	133, 100 (0.00- 0.00)	155, 99.34 (98.09- 100.00)
Upper respiratory infection			
Yes	36, 12.24 (8.48- 16.01)	16, 11.76 (6.28- 17.25)	20, 12.66 (7.42- 17.90)
No	258, 87.76 (83.99- 91.52)	120, 88.24 (82.75- 93.72)	138, 87.34 (82.10- 92.58)
Living with twin/sibling			
Yes	159, 45.73 (48.53- 60.00)	80, 59.26 (50.86- 67.65)	79, 50 (42.12- 57.88)
No	134, 45.73 (40.00- 51.47)	55, 40.74 (32.35-49.14)	79, 50 (42.12- 57.88)
Sharing room with twin sibling			
Yes	84, 28.67 (23.46- 33.88)	47, 34.81 (26.68- 42.95)	37, 23.42 (16.74- 30.09)
No	209, 71.33 (66.12- 76.54)	88, 65.19 (57.05- 73.32)	121, 76.58 (69.91- 83.26)

From 294 twin participant samples, 178 sites (178/882, 20.2%) were positive for *S. aureus* via culture-dependent techniques. Overall, carriage for the nares, oropharynx, and hand were 26.2%, 29.9%, and 4.4%, respectively. For 43 participants, *S. aureus* was restricted to the nares, 52 were restricted to the oropharynx, and 4 were restricted to the hand. Twenty-eight participants were co-colonized in the nares and oropharynx, three co-colonized in the oropharynx and on the hand, and one participant was co-colonized in the nares and on the hand. Five participants were colonized at all three sites (nares, oropharynx, and hand).

### Concordance

We calculated concordance as the number of twin pairs that were concordant positive for *S. aureus* divided by the cumulative number of twin pairs that were either concordant or discordant positive. Among monozygotic twin pairs, a total of 25 (25/54; 46.3%) twin pairs were concordant culture-positive for *S. aureus* and 29 (29/54; 53.7%) were discordant within the anterior nares (**Table 2**). Within oropharynx culture-positive samples, a total of 19 (19/69; 27.5%) twin pairs were concordant culture-positive for *S. aureus* whereas 50 (50/69; 72.5%) were discordant. Only 1 (1/12; 8.3%) set of twins were concordant for *S. aureus* carriage in the hand samples with 11 (11/12; 91.7%) twin pairs being discordant. A Chi-square analysis revealed significant variation in concordance patterns across the three sites. In the nares, concordance showed a strong and statistically significant association (χ2 = 34.33, p<0.001). The oropharynx showed a moderate but statistically significant association (χ2 = 4.751, p= 0.029), while no significant concordance was observed in the hand samples (χ2 = 0.176, p=0.675).

**Table 2:** Concordance of *Staphylococcus aureus* carriage in monozygotic twin pairs in the nares, oropharynx, and on hands. Each number represents a twin pair comparison.

**Nares**				
			**Twin A**	
			Positive	Negative
	**Twin B**	Positive	25	12
		Negative	17	93
**Hand**				
			**Twin A**	
			Positive	Negative
	**Twin B**	Positive	1	5
		Negative	6	135
**Oropharynx**				
			**Twin A**	
			Positive	Negative
	**Twin B**	Positive	19	20
		Negative	30	78

### Alpha and beta diversity

**Figure 1:**
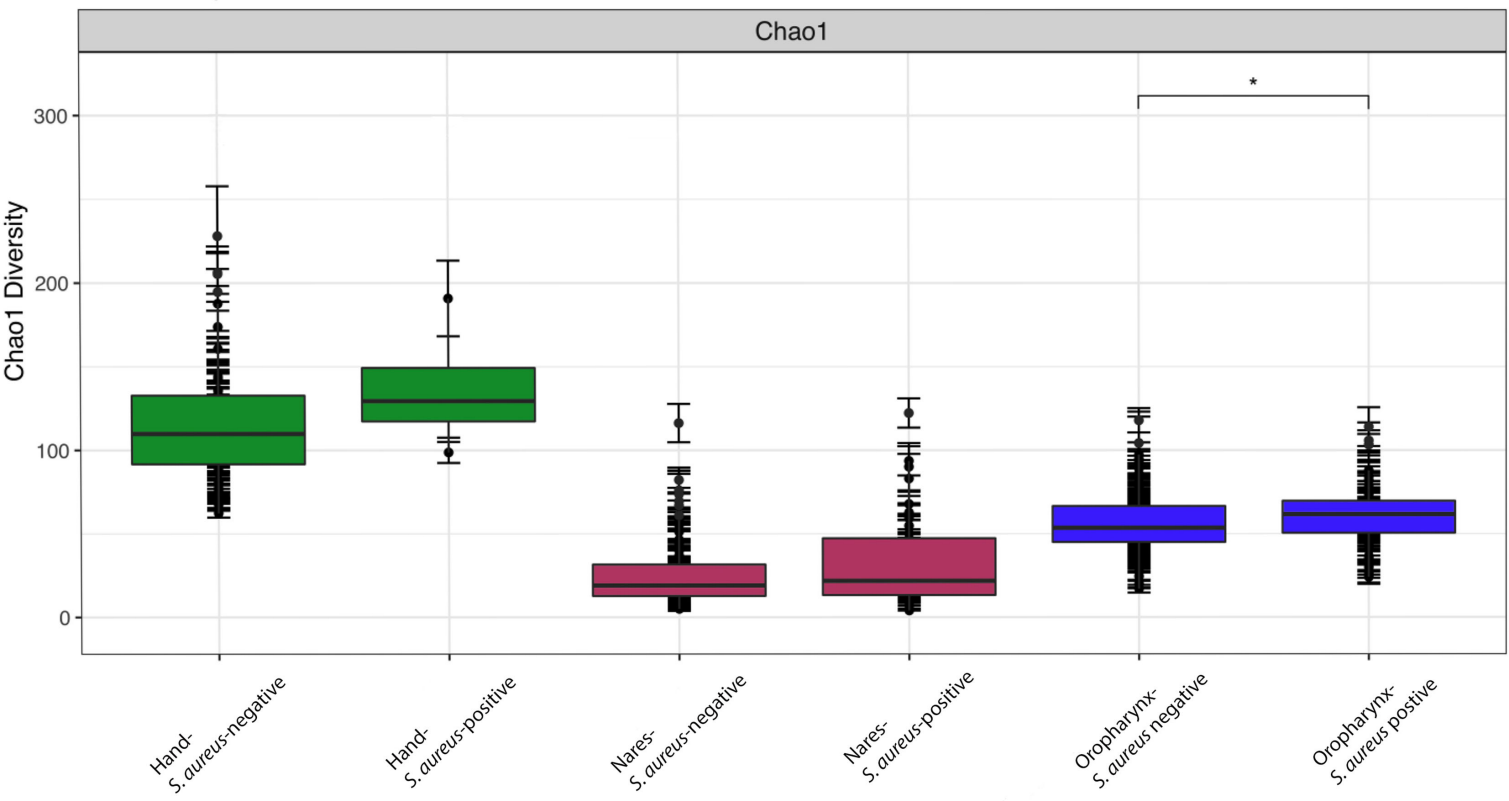
Chao1 alpha diversity indices indicating community species richness for the nares, oropharynx, and hand samples, separated by *S. aureus* culture status. Data are presented as mean ± SD with median and interquartile ranges shown. All site comparisons were significant at p ≤ 0.05. Within the oropharynx samples, only culture-positive versus culture-negative sites was significantly different (*p = 0.05).

We obtained 4,680,299 high-quality filtered reads across 1,351 ASVs. Sample sequencing depth ranged from 2,527 to 46,685 reads with an average of 10,589 reads. We rarefied all samples to an even sampling depth of 2,527 reads. Alpha diversity was calculated to measure the richness of community microbial species within each sample. The Chao1 diversity (**Figure 1**) was greatest in the hand samples, regardless of *S. aureus* culture status (p <0.001) when compared to other sites (nares and oropharynx) and their corresponding culture status (positive/negative). There was no significant difference in the Chao1 diversity between hand culture-positive and culture-negative samples (p>0.05). The oropharynx exhibited the second-highest alpha diversity of the three sites sampled. The nares had the lowest alpha diversity of all sites sampled, with no significant difference in alpha diversity between culture-positive nares and culture-negative nares (p> 0.05). The Chao1 alpha diversity was the only index tested of three (Shannon and Simpson index figures can be found in **Supplementary Data Sheet 2**) to find a significant difference between culture-positive and culture-negative oropharynx samples (p=0.05). Diversity did not differ between culture statuses within the nares nor hand sites (p >0.05). There were, however, significant differences in alpha diversity when comparing collective nares to oropharynx, oropharynx to hand, and nares to hand samples (**Figure 1**; p<0.001).

**Figure 2:**
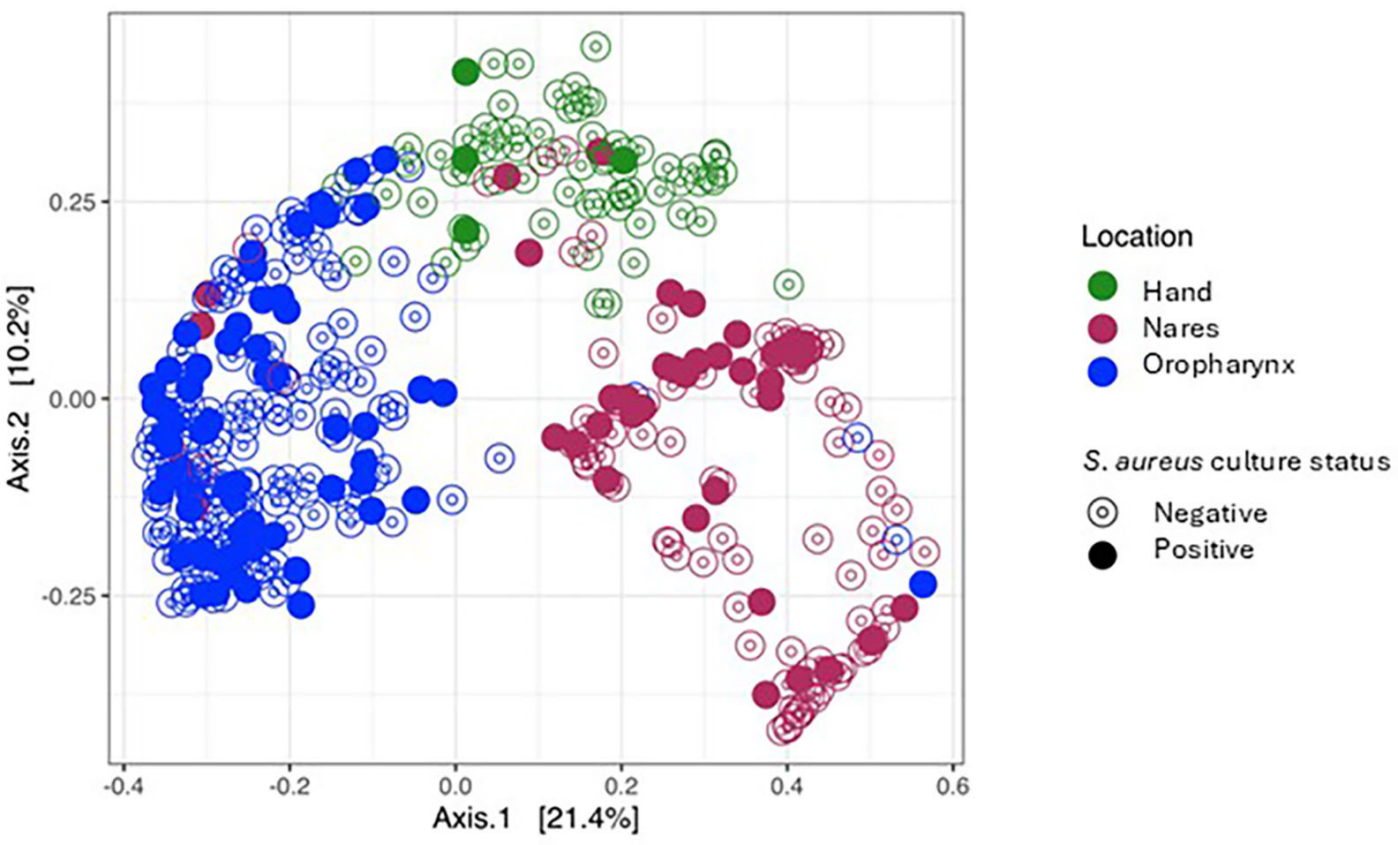
Principal Coordinates Analysis (PCoA) of monozygotic twin participants by location and *Staphylococcus aureus* culture-dependent status (Bray–Curtis).

**Figure 3:**
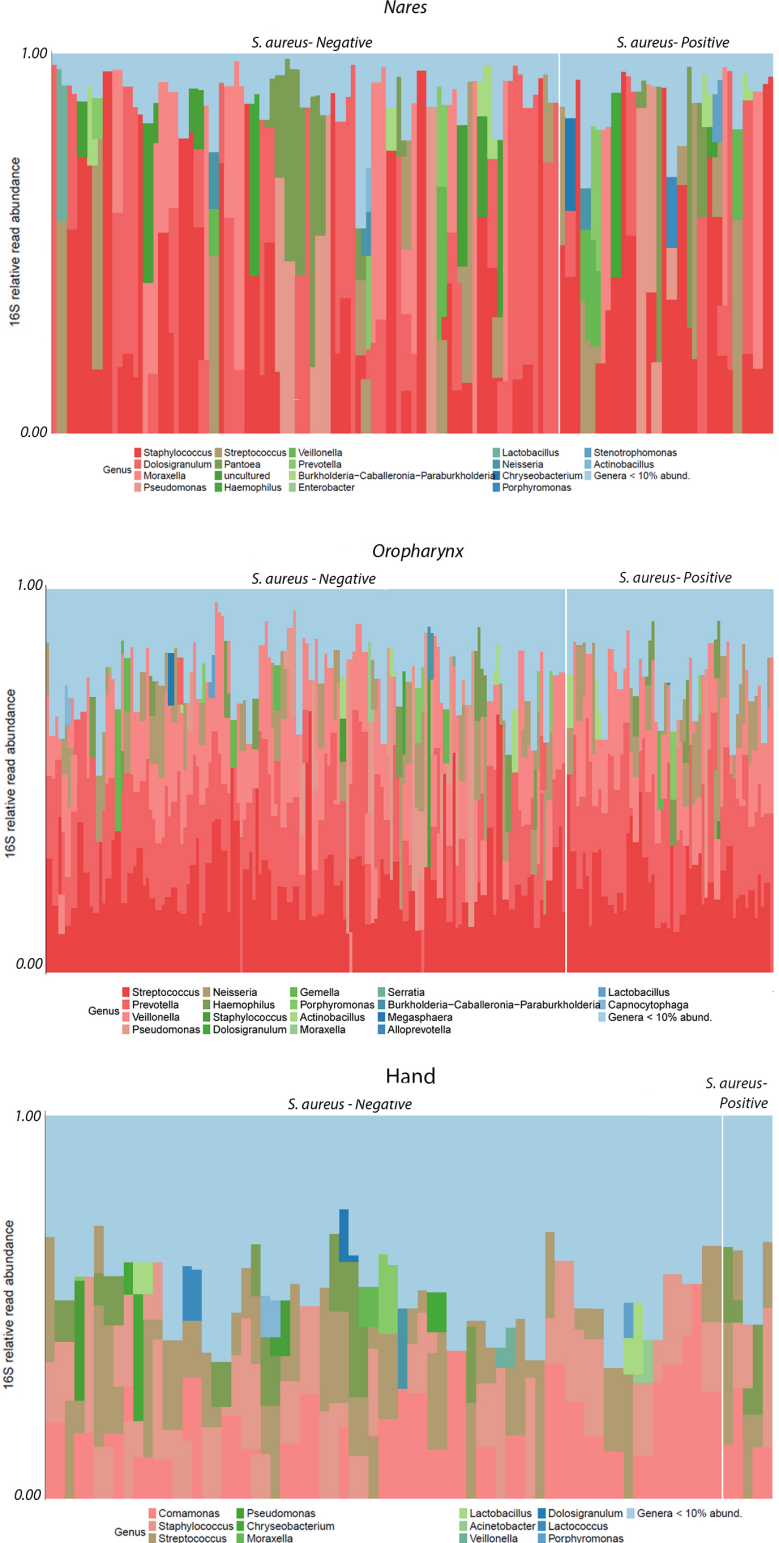
16S Relative Read Abundance (RRA) of monozygotic twin samples as a function of location (nares, oropharynx, and hand) and culture-dependent status (*S. aureus* positive/negative) plotted at the genus level.

Beta diversity was calculated to measure the diversity across all samples using the Bray–Curtis method (both weighted and unweighted UniFrac were also calculated and can be found in **Supplementary Data Sheet 3**). The ordination plot of the Bray–Curtis distances for all samples is shown in **Figure 2**. The samples primarily clustered by sample location (nares, oropharynx, and hand; a PERMANOVA was conducted to test for differences in mean and variance for the groups’ centroids, p= 0.001) but also by *S. aureus* culture status (p= 0.001). Additionally, the interaction between variables, location, and culture was also highly significant (p= 0.001). To test what was driving the differences in beta diversity, a PERMDISP function was run to test differences in dispersion (variance) between any of the groups. As shown in **Figure 3**, the beta dispersion of the locations was highly significant (p=0.001) whilst the dispersion of the *S. aureus* culture status of samples was not significant (p=0.151) and was not driving the observed beta diversity.

### Relative read abundance (%)

The RRA of taxa at the phylum and class levels showed no observable differences between culture statuses across location sites (data not shown). However, descriptive differences in RRA were noted between locations (hand, nares, and oropharynx) at the order and genus levels (**Figure 3**). The most pronounced variations in RRA between culture-positive and culture-negative samples within locations were observed at the order and genus levels, with more taxa identified in culture-negative samples from the nares, oropharynx, and hand. A total of six bacterial orders were found in the nares, with *Burkholderiales* being enriched in the *S. aureus*-negative samples. In both hand and oropharynx culture-negative samples, there was enrichment of *Pseudomonales* compared to their respective culture-positive samples at the order level. Additionally, the oropharynx was the only site (compared to nares and hand) to have increased RRA of *Veillonellales (Selenomonadales)* and *Bacteroidales* in their samples. At the genus level, the nares had the highest RRA of all sites with almost 50% (0.48 16S RRA) of *Staphylococcus* in culture-positive samples. The second most abundant in the nares was the genus *Dolosigranulum*. The most dominant genus on the hands was a collective number of genera with <5% abundance making up more than 30% of the composition in both culture-positive and -negative hand samples. The next most abundant in the hand samples were *Prevotella*, *Comamonas*, and *Staphylococcus* in that order. In the oropharynx, *Streptococcus* was the most abundant genera followed closely by *Prevotella* and *Veillonella*.

### Querying shared microbiota composition between monozygotic twins

**Figure 4:**
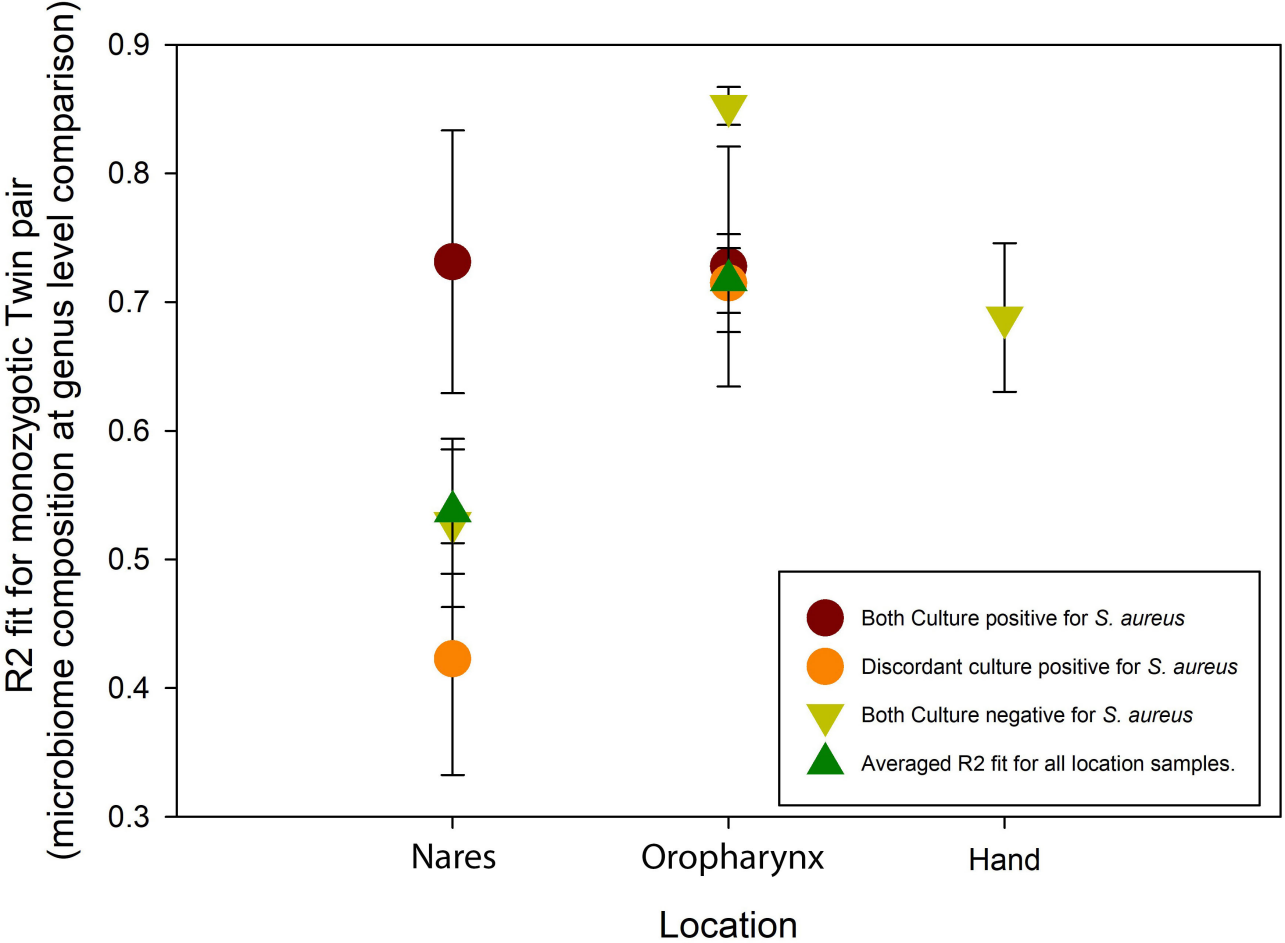
R^2^ fit of microbiome composition results between monozygotic twin pairs (analyzed at the genus level). Data are plotted as mean± SEM.

In the nares, concordant culture-positive twins exhibited greater similarity in bacterial genera composition (R² = 0.73 ± 0.10) than concordant culture-negative twins (R² = 0.53 ± 0.65), with discordant twins showing the lowest similarity (R² = 0.42 ± 0.09) (**Figure 4**). In the oropharynx microbiome, concordant culture-positive twins (R² = 0.73 ± 0.09) and discordant culture-positive twins (R² = 0.72 ± 0.04) displayed comparable levels of bacterial genera similarity. Notably, concordant culture-negative twins had the highest similarity in the oropharynx microbiome (R² = 0.85 ± 0.02). Although data for the hand microbiome was limited, culture-negative twin pairs showed a distinct similarity in bacterial composition (R² = 0.69 ± 0.06).

### Differentially abundant taxa

**Figure 5:**
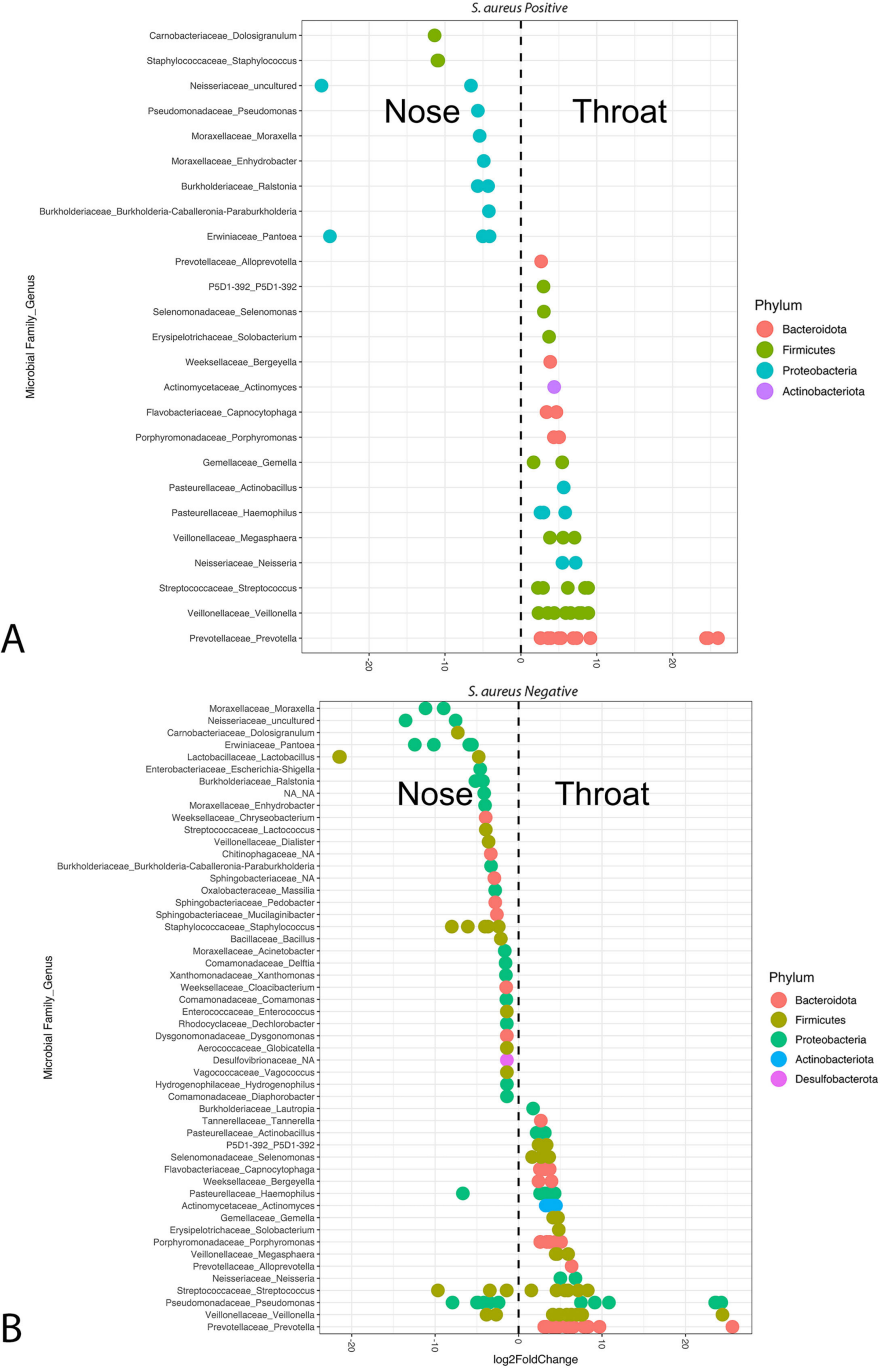
Differential abundance of bacteria taxa using DeSeq2 from **(A)** *S. aureus* culture-positive and **(B)** *S. aureus* culture-negative nares and oropharynx samples. These plots show the microbial taxonomy at the family and genus level (y-axis). Multiple points along a single y-axis row indicate that multiple microbial ASVs were differentially abundant within that family and genus level, representing different species or variants within the genus.

The DESeq2 method (log(Count +1) transformed data) was used to identify differentially abundant taxa across locations (nares, oropharynx, hand) and *S. aureus* culture status (positive vs. negative) (**Figure 5**). In nares samples, *Moraxella nonliquefaciens* (*Proteobacteria*) was enriched in culture-negative samples (padj = 0.0065), while *Capnocytophaga* (*Bacteroidota*) was enriched in culture-negative oropharynx samples (padj = 0.0032). No significant differences were observed in hand samples between culture statuses.

A total of 61 genera were significantly enriched between culture-positive samples from the nares and oropharynx (**Figure 5A**). In the nares, nine unique genera, all within *Proteobacteria* or *Firmicutes*, were significantly enriched compared to the oropharynx, with uncultured *Neisseriaceae*, *Pantoea*, *Dolosigranulum*, and *Staphylococcus* as the most abundant (padj < 0.001). In contrast, 16 unique genera were enriched in the oropharynx compared to the nares, with *Prevotella* as the most significantly enriched genus, followed by *Veillonella* and *Streptococcus* (padj < 0.001 each). Notably, the oropharynx microbiome showed diversity across four phyla—*Bacteroidota*, *Firmicutes*, *Proteobacteria*, and *Actinobacteria*—while the nares were primarily enriched with *Firmicutes* and *Proteobacteria*.

Among culture-negative samples from the nares and oropharynx, 145 genera were significantly enriched, with 52 unique genera shown in **Figure 5B**. In the nares, four phyla (*Bacteroidota*, *Firmicutes*, *Proteobacteria*, and *Desulfobacterota*) were enriched, with 64 genera (33 unique) showing significance; *Lactobacillus* was the most enriched (padj < 0.001), followed by uncultured *Neisseriaceae*, *Staphylococcus* (-6.08 ± 1.29 log2FoldChange; padj < 0.001), and *Escherichia*-*shigella* (padj < 0.001). Conversely, oropharynx samples had 81 significantly enriched genera (19 unique), with *Prevotella*, *Veillonella*, *Pseudomonas*, *Streptococcus*, and *Actinomyces* as the most abundant (padj < 0.001 each).

## Discussion

*S. aureus* is a common commensal of the human microbiome, and its carriage is a significant risk factor for severe invasive infections with high mortality. Understanding factors that are associated with *S. aureus* carriage may lead to strategies for preventing these infections. Previous traditional twin studies have suggested that host genetics play a role in *S. aureus* carriage within the microbiome, though the degree of genetic influence remains debated (Andersen et al., 2012; Cyr et al., 2017; Peacock et al., 2001). Studies on the gastrointestinal microbiome have similarly identified a genetic contribution to microbiome composition (Goodrich et al., 2016; Turnbaugh et al., 2009; Vilchez-Vargas et al., 2022) though these effects seem to be in microbiomes less impacted by the environment. Using an observational approach in monozygotic twins, we investigated *S. aureus* concordance and microbiome composition across the nares, oropharynx, and hand. Our analysis revealed that location was the most significant modifier of microbiome composition while *S. aureus* carriage was significantly associated with microbial diversity and composition in monozygotic twins, albeit not similarly across all sampled locations.

### Concordance

We characterized the microbiome composition of the nares, oropharynx, and hand of 147 monozygotic twin pairs, and described the compositional association of *S. aureus* culture status. Our study demonstrates that *S. aureus* culture status does associate with distinct microbiome composition in genetically identical twin pairs and that despite *S. aureus* concordance rates between MZ pairs ranging from 8%-46% at the three sites sampled, microbiome similarity was highest for concordant pairs. Microbiome composition was more similar between CC *S. aureus* pairs than DC *S. aureus* pairs (at the genus level) in both the nares and oropharynx [R² = Nares 0.73 (CC) vs 0.42 (DC); Oropharynx 0.85 (CC) vs 0.72 (DC)]. A recent study on Korean monozygotic twins also found that MZ twins’ microbiomes are more similar than DZ twins (Si et al., 2015) and our study reaffirms this finding while also noting that discordance for *S. aureus* carriage resulted in the least similar microbiome composition in monozygotic twin pairs at the genus level.

Our twin concordance rate for *S. aureus* positivity in the nares (46.3%) was notably higher than concordance rates for autoimmune conditions in Finnish twins, such as type 2 (34%) and type 1 diabetes (23%) (Kaprio et al., 1992), as well as *S. aureus* concordance rates reported in previous nasal microbiome studies by Andersen et al. (37.5%, 2012) and Liu et al. (2015) (26.1%, 2015). However, it remains lower than rates associated with conditions influenced by microbial presence, such as Crohn’s disease, inflammatory bowel disease, and ulcerative colitis (approximately 50%; Jess et al., 2005). Concordance in the oropharynx was 27.5%, while the hand exhibited a lower concordance of 8.2%, consistent with findings that suggest the hand microbiome is more variable and likely environmentally driven (Edmonds-Wilson et al., 2015). Notably, the oropharynx microbiome showed the highest genus-level similarity among concordant twins, with similarity strongest among twin pairs who were both non-colonized by *S. aureus* (R² = 0.85), suggesting that *S. aureus* colonization may increase microbiome variability in the oropharynx more than in the nares. Similar microbiome shifts impacting alpha diversity have been observed in other vertebrates (Barbagelata et al., 2011; Khamash et al., 2018; Park et al., 2011). Overall, our *S. aureus* concordance data from strictly monozygotic twins suggest an intermediate position, aligning with traditional twin studies that indicate minimal genetic influence (Andersen et al., 2012; Roghmann et al., 2011) but also supporting findings of modest genetic contributions to concordance rates (Aly et al., 1974; Hoeksma and Winkler, 1963; Si et al., 2015).

### Prevalence

The overall prevalence of *S. aureus* we identified in the nares, oropharynx, and hand (26.2%, 29.9%, and 4.4%, respectively) aligns with prior studies showing approximately 30% of the general population are carriers (Gorwitz et al., 2008; Sollid et al., 2014). However, our rates were lower than those reported for medical students (39.3%; López-Aguilera et al., 2013), healthy Midwestern adults and children (44.1% and 36.1%, respectively; Hanson et al., 2018), and young women not using hormonal contraceptives (34%; Stensen et al., 2019). Although this study did not include longitudinal sampling, studies from Mexico reported higher *S. aureus* carriage (59.3%) across the nares and oropharynx (Hamdan-Partida et al., 2010). When comparing oropharynx to nares prevalence, our study observed a higher carriage rate in the oropharynx, similar to findings in prison populations (42.7% vs. 35.0%; Lee et al., 2011), healthy Iowans (37.9% vs. 25.4%; Hanson et al., 2018), and orthopedic patients (40% vs. 31%; (Nilsson and Ripa, 2006), but opposite to young children, where nares prevalence was higher (25.9% vs. 39.2%; (Esposito et al., 2014). Interestingly, *S. aureus* prevalence on the hand was markedly lower than in studies of food handlers (28.7%; (Beyene et al., 2019) and closer to healthcare worker rates (10.5%; Tammelin et al., 2003). Although we did not control for the time since the last handwashing (which could influence hand carriage rates), previous studies have shown that post-decolonization time can affect *S. aureus* prevalence and may explain the lower rates observed on hands in our study (Beyene et al., 2019).

### Differential biodiversity

When comparing biodiversity within (alpha) and between (beta) sampled sites differentially colonized by *S. aureus*, we found that only the oropharynx site showed a significant difference in alpha diversity between *S. aureus* culture-positive and culture-negative samples (Chao1 index, p=0.05), using an index suited for low-abundance studies (Kers and Saccenti, 2022). No significant differences were observed using the Shannon and Simpson diversity indices (p>0.05; **Supplementary Data Sheet 2**). In contrast, other studies have reported that oropharynx alpha diversity can vary significantly over time (Bach et al., 2021), and although we sampled only healthy participants, Zhao et al. (2021) found that low alpha diversity is linked with increased *S. aureus* bacteremia in ICU neonates, suggesting that *S. aureus* presence may impact microbiome composition. Comparing alpha diversity across sites (nares, oropharynx, and hand), significant differences emerged (p<0.01; **Figure 2**), supporting prior findings that skin microbiome environments differ by site (Perez et al., 2016). Overall, the hand exhibited the highest alpha diversity, while the nares had the lowest (**Figure 2**), consistent with studies showing variability in alpha diversity across human ecological niches, including nares, oropharynx, sputum, feet, and skin (De Boeck et al., 2017; Han et al., 2020; Kates et al., 2019). Given that hand microbiome composition is known to vary widely between individuals and is influenced by factors such as sex (Basic-Cicak and Hasic Telalovic, 2023; Edmonds-Wilson et al., 2015), our findings of higher alpha diversity on the hand relative to the nares and oropharynx were not unexpected.

Biodiversity across sampling sites was measured using Bray–Curtis distance to assess taxon-based compositional differences in the nares, oropharynx, and hand samples based on *S. aureus* culture status. PCoA plots supported the hypothesis that *S. aureus* presence modifies beta diversity (p=0.001) and interacts with sampling sites (p=0.001). Previous twin studies found no significant differences in alpha or beta diversity in conditions such as rosacea (Zaidi et al., 2018) or the skin microbiome of Korean twins (Si et al., 2015). However, other studies have reported inverse relationships between *S. aureus* colonization and other microbiota (Bessesen et al., 2015) or even increased biodiversity due to *S. aureus* colonization (Reyes et al., 2020). To further clarify, while *S. aureus* status contributes to statistical differences in beta diversity, these findings may not represent biologically meaningful shifts in composition across all body sites. Despite this, our data do support the hypothesis that *S. aureus* carriage influences beta diversity, reflected in increased RRA at the family level: *Neisseriaceae* and *Staphylococcaceae* on the hands; increased *Staphylococcaceae* and low-abundance families in the nares for *S. aureus* culture-positive individuals; increased *Carnobacteriaceae* in culture-negative participants; increased *Prevotellaceae* and low-abundance families in *S. aureus* culture-positive oropharynx samples; and increased *Pseudomonadaceae* in culture-negative oropharynx samples. Increased *Neisseriaceae* abundance has been linked to chronic rhinosinusitis and *S. aureus* carriage (Wagner Mackenzie et al., 2021). *Carnobacteriaceae*, found in low abundance in other nasal studies (5.7%; (Lu et al., 2018), shows an inverse relationship with *S. aureus* (Flores Ramos et al., 2021), supporting our findings in the culture-negative samples. Additionally, our study found increased *Prevotellaceae* abundance correlated with *S. aureus*, consistent with findings in MRSA skin and soft tissue infections (SSTI) lesions (Misic et al., 2015), though our study did not assess MRSA SSTIs. Interestingly, the increased *Pseudomonadaceae* abundance in culture-negative participants aligns with other research suggesting that *P. aeruginosa* can outcompete *S. aureus* in wounds (Yung et al., 2021), despite reports of synergistic effects in other contexts (Alves et al., 2018; DeLeon et al., 2014).

To identify differentially abundant taxa, we used DESeq2 (Calgaro et al., 2020). In the nares, only one species, *M. nonliquefaciens*, was significantly more abundant in samples negative for *S. aureus*. Other studies have similarly reported *Moraxella* species in healthy controls (van den Munckhof et al., 2020), noting that *Moraxella* acts independently of *S. aureus* in the upper respiratory tract (Claassen-Weitz et al., 2021). However, *M. nonliquefaciens* can also cause infections similar to *S. aureus*, such as botryomycosis, a chronic granulomatous skin disease (Feldman and Petersen, 1989). In the oropharynx, only the genus *Capnocytophaga* was significantly more abundant in *S. aureus* culture-negative monozygotic twins. Known as a common oral commensal in humans and pets, *Capnocytophaga* supports oral health (Aas et al., 2008; Gross et al., 2010; Sixou et al., 2006) but has been linked to severe endocarditis in humans (Hayani et al., 2009; Sakai et al., 2019), similarly to *S. aureus* (Baddour et al., 2015). No differentially abundant taxa were observed between culture-positive and culture-negative hand samples.

When comparing differentially expressed taxa between the nares and oropharynx in *S. aureus* culture-positive samples, the most abundant taxa in the nares included Dolosigranulum, *Staphylococcus*, and *Pantoea*, while *Prevotella*, *Streptococcus*, *Neisseria*, and *Alloprevotella* were more notable in the oropharynx. Studies have shown that *Dolosigranulum* is commonly found in the nares (De Boeck et al., 2017) and is inversely associated with *S. aureus* carriage (Brugger et al., 2020). This inverse relationship is reflected in our data, as *Dolosigranulum* was significantly more abundant in the nares than *Staphylococcus*. Additionally, the presence of *Neisseriaceae* in the oropharynx aligns with studies showing their frequent co-occurrence (Drayß et al., 2019; Weyand, 2017), while *Pantoea* has also been identified in the nasal-oropharyngeal region (Walterson and Stavrinides, 2015). Similarly, co-presence of *S. aureus* and *Prevotella* has been observed in foot and hand paronychia (Grande-Del-Arco et al., 2020) and murine MRSA pneumonia models (Yamashita et al., 2020).

When evaluating differentially abundant taxa between *S. aureus* culture-negative and culture-positive nares and oropharynx samples, more abundant taxa were observed in *S. aureus* culture-negative samples. For instance, the most prevalent taxa in culture-negative nares were *Lactobacillus*, *Moraxella*, and *Ralstonia*, all typically found in the oropharyngeal microbiome. In the absence of *S. aureus*, these taxa may be more prominent in the nares. *Lactobacillus*, particularly strains with catalase function, is known to inhabit healthy nasal microbiomes (De Boeck et al., 2017), while *Moraxella* is frequently observed in healthy elderly individuals (van den Munckhof et al., 2020). *Ralstonia*, though commonly a soil microbe, appears in patients under mechanical ventilation (Waugh et al., 2010) and has a symbiotic relationship with *Rhizopus*, enhancing virulence and resistance to phagocytosis (Itabangi et al., 2022). Conversely, in the *S. aureus* culture-negative oropharynx samples, *Haemophilus*, *Prevotella*, and *Streptococcus* were among the most abundant taxa. Studies have shown *Haemophilus* can support *S. aureus* attachment on surfaces (Esin et al., 2015), potentially aiding carriage, though both taxa are sometimes found together (Nair et al., 2014). As noted previously, *Prevotella* was common in both *S. aureus* culture-positive and -negative oropharynx samples, suggesting that its abundance is influenced by location rather than *S. aureus* presence. The increased abundance of *Streptococcus* in *S. aureus* culture-negative oropharynx samples aligns with studies showing *Streptococcus* can inhibit *S. aureus* through hydrogen peroxide activity (Bogaert et al., 2004; Regev-Yochay et al., 2006), though *S. aureus* also modifies streptococcal biofilms (Schnurr et al., 2021), with antioxidants potentially affecting both taxa in biofilms (Sempere et al., 2022).

## Limitations

This study has some limitations that could be addressed in future research. First, this study only investigated monozygotic twins. Although this minimized genetic variability, disentangling genetics from environment would require the addition of dizygotic twins to the study design and even investigating twins reared apart. Second, we did not account for the time twins spent together, which may influence *S. aureus* transmission via shared surfaces. Next, molecular typing was not performed, meaning concordant twins could carry two distinctly different *S. aureus* strains, potentially driving microbiome differences beyond simple presence or absence. Fourth, as a point-prevalence study, we lack longitudinal data. Although the International Twin Festival setting restricted repeated sampling, longitudinal data would provide clearer insights into the dynamics of *S. aureus* carriage over time. Additionally, we did not investigate the mechanistic impact of *S. aureus* carriage on microbiome composition or manipulate its composition. Despite these limitations, the study’s strengths include a large sample size and extensive sampling per participant, which enhances our understanding of *S. aureus* carriage associations within monozygotic twin pairs.

## Conclusions

Our findings support our first prediction that *S. aureus* carriage is associated with reduced microbial diversity, as indicated by RRA and DESeq2 analyses showing fewer abundant taxa, potentially due to competitive exclusion by *S. aureus*. Specifically, in the *S. aureus*-negative samples, *M. nonliquefaciens* and *Capnocytophaga* were enriched in the nares and oropharynx, respectively, suggesting that these taxa may be outcompeted in *S. aureus*-positive environments.

Chao1 diversity narrowly achieved significance in only the oropharynx samples, pointing to a subtle yet detectable deviation from our hypothesis. Consistent with our second hypothesis, monozygotic twin pairs showed the highest concordance for *S. aureus* colonization in the nares, followed by the oropharynx and hand. STAMP analyses further revealed that microbiome composition was most similar in the *S. aureus*-positive nares samples, while the *S. aureus*-negative oropharynx samples showed an inverse pattern, likely reflecting influences of sampling location and/or timing. Bray–Curtis beta diversity analysis confirmed that location was the main factor shaping microbiome composition, with *S. aureus* status introducing additional, site-specific variation that acted as a modifier to compositional differences.

## Data Availability

The datasets presented in this study can be found in online repositories. The names of the repository/repositories and accession number(s) can be found in the article/**Supplementary Material**.
