# Supplementary material for: Staphylococcus aureus carriage is associated with microbiome composition in the nares and oropharynx, not the hand, of monozygotic twins
Source: Front Microbiomes. 2025 Jan 20;3:1457940. doi: 10.3389/frmbi.2024.1457940 (PMC12993631; doi:10.3389/frmbi.2024.1457940)
Supplement: Supplementary file 1 [file DataSheet1.pdf]

KENT STATE UNIVERSITY College of Public Health

Smith Emerging Infections Lab

Background Demographics and health

1.Todays date______/______/___________ (month/ day/ year XXXX)

2. Date of Birth ______/______/___________ (month/ day/ year XXXX)

3. Sex: Male Female

4. What is your height: _____(feet) _____ (inches) and weight__________ (lbs)

5. What is your ethnicity ______________________

6. Marital status (circle): Married Divorced Single Separated Widowed

7. Family size (individuals currently living in your house, including yourself) ____________________
   7a. How many children under the age of 18 currently living in the household_________
   7b How many are enrolled in a daycare?_____________________________________

8. Do you currently live with your (circle): sister brother
   8a. Do you share your room with your sibling (s).

9. Were you born (please circle one)? Caesarian or natural

10. What was your birth weight _____________________(lbs) and week of birth ___________week

11. What was the last grade you completed in school?
   High school graduate
   Some college
   College graduate
   Postgraduate/ professional
   Other (specify) ____

12. As a child, did you receive all of your doctor recommended vaccines?
   YES
   NO
   Don't know or don't remember

13. Which hand do you use more often? (select one)
   Right
   Left
   Neither, I use both hands equally

14. Do you currently have any of the following conditions? (circle all that apply)

Asthma
Emphysema
Chronic obstructive pulmonary disorder
Heart disease or diseases of your blood vessels, veins or arteries
Diabetes (type 1 or 2)
Kidney disease
HIV/ AIDS
Food Allergies
Autoimmune disease (specify)____

15. Have you been diagnosed with cancer in the last five years?

Yes ____
Yes, in remission (year?)____
No, in remission (year?) ____
No

16. Do you have any condition that may weaken the immune system?

Yes____
No____
I don't know

17. Do you take medications such as anti-cancer drugs, sterioids (such as prednisone). Or other drugs that weaken the immune system in the last 3 months?

Yes
No

18. Have you been diagnosed with an upper respiratory tract infection, ear infection, or sinus infection in past 3 months?

Yes
No

19. Have you taken antibiotics in the past 3 months?

Yes
No

20. Have you had the nasal influenza (flu) vaccine, sometimes called FluMist of nasal flu spray, in the past 3 months?

Yes
No

21. Have you ever been told by a doctor that you have any of the following skin conditions (check any/ all that apply)?

Eczema
Psoriasis
Folliculitis
Red bumps or pimples
Other____

22. Have you had a skin or soft tissue infection (such as an infection of the muscle, abscess, furuncle, impetigo) in the past 3 months?

Yes (if yes proceed to question 22a)
No

22a. Where on your body was the infection? (circle all that apply)

Legs	Stomach/ waist
Arms	Groin
Hands	Back
Face	

23. Have you been told by a doctor you have a staph infection in the previous 3 months?

Yes
No
I don't know

24. Have you been told by a doctor you have a MRSA (methicillin-resistant Staphylococcus aureus infection in the past 3 months?

Yes
No
I don't know

25. Have you used any type of probiotics in the past 3 months?

Yes (specify)____
No
I don't know

26. How often do you brush your teeth? (Please circle your answer)

Once Daily
Twice daily
After every meal
I don't brush my teeth

Home environment

27. Do you currently have pets?

No
Yes (if yes specify by circling below types)

- Dogs
- Cats
- Reptiles (turtle, snake, lizard, iguana, etc)
- Small mammal (mouse, rabbit, guinea pig, gerbil, etc)
- Bird
- Fish
- Other (specify)____

28. Do you have a water softener in your home?

Yes
No
I don't know

29. What type of water do you have in your home?

Well water
Public water
Don't know

Hygiene

30. How often do you shower/ take a bath?

Every day
Every other day
once a week
less than once a week

31. Do you wash your hands before eating?

No
Yes

32. What types of hand soaps are used in your home (check all that apply)?

Bar soap, not antibacterial (Dove, Ivory, etc)
Bar soap, antibacterial (Dial, Lever 2000, etc)
Liquid soap, not antibacterial
Liquid soap, antibacterial
Other (describe:________________________________)

33. Which statement best describes the skin on your hands? (pick one)

My hands are very healthy. They look and feel good. I rarely suffer from redness, dryness, crackling, or stinging

My hands are healthy. They look and feel good most of the time. Occasionally get red, dry, or hurt.

My hands are OK, but not great. Sometimes they look and feel good, but other times are red, dry, cracked, or sting.

My hands are in bad condition. They rarely look or feel good. I almost always suffer from redness, dryness, cracking, or stinging.

34. Are bath towels shared in your household without being washed in between users?

Yes

No
I don't know

35. Are hand towels shared in your household without being washed in between users?
Yes
No

36. How do you feel about overall hygiene? (pick one)
I'm very adamant about washing my hands and cleaning my home and making sure I live in a bacteria free environment

I try to keep a clean home and I'm very aware of my personal hygiene. I do what I can to keep myself and my home tidy

Sometimes I feel like people go a little overboard with sanitizing and cleaning. I feel like dirt and bacteria serve a purpose and help us build our immune system

Proceed to next section if under 18 years old

37. Are you currently changing diapers?
Adult diapers
Yes
No
Child diapers
Yes
No

Outdoor activities

38. In the last month, how much time do you spend gardening, landscaping, or other activities that involve touching dirt?
Less than 1 hour per week
1-5 hours per week
5-10 hours per week
More than 10 hours per week

39. As a child, did you play more indoors or outdoors?
Indoors
Outdoors
I spent equal time indoors and outdoors
Don't remember

40. In the last month, how much time are you spending outdoors each week?
Less than 1 hour per week
1-5 hours per week
5-10 hours per week
More than 10 hours per week

41. How often do you use a swimming pool or hot tub?

Almost everyday
Weekly
Every few weeks
Monthly
A couple times a year
Never

42. Have you participated in team and/or contact sports in the last 3 months?

Yes
No

43. In the past month, how often have you attended a gym or community workout facility?

Zero
1-3 times a week
4-6 times a week
7 or more times a week

Food exposure

44. Do you have any dietary restrictions?

Yes (specify)____
No

45. How often do you eat the following foods (check one per row)?

	DAILY	WEEKLY	RARELY
fast food			
eggs			
red meat			
lean meat (ie. Turkey, fish, chicken)			
fruit			
vegetables			
sweets			
milk products (ie. Yogurt, cheese)			
raw/rare meat, poultry, fish or eggs; sushi; unpasteurized dairy			

46.. Check boxes to show what products your hands have come in contact with **in the past 30 days** and about how often. **Include those that you apply directly to your hands as well as products you *use your hands to apply elsewhere*.**

Product	Multiple times a day	Once a day	Once a week	Less than once a week	Have Not used
hand lotion					
body lotion					
face lotion					
plain hand soap (non-antimicrobial)					
anitmicrobial hand soap					

Product	Multiple times a day	Once a day	Once a week	Less than once a week	Have Not used
body soap / body wash					
hand sanitizer					
baby wipes					
antimicrobial hand wipes					
antimicrobial surface wipes					
antimicrobial surface cleansers					

Product	Multiple times a day	Once a day	Once a week	Less than once a week	Have Not used
dish washing detergent					
sunscreen					
shampoo					
conditioner					
hair styling products					

Product	Multiple times a day	Once a day	Once a week	Less than once a week	Have Not used
shaving cream					
baby rash cream					
acne medication					
anti-aging products					
makeup					

47. Listed below are statements about hygiene and health. For each statement pick whether you Strongly agree, Agree, Neither agree or disagree, Disagree, or Strongly Disagree

	Strongly agree	Agree	Neither agree or disagree	Disagree	Strongly disagree
I make sure my vaccinations and those of my children are up to date.	5	4	3	2	1
I only use hand sanitizer in public places, not in my home.	5	4	3	2	1
I work out at the gym regularly (at least 2x per week)	5	4	3	2	1
I play team sports.	5	4	3	2	1
I purchase cleaning products with a short list of ingredients that I recognize	5	4	3	2	1
	Strongly agree	Agree	Neither agree or disagree	Disagree	Strongly disagree
I would say that I clean / disinfect my house more than the average person.	5	4	3	2	1
I eat yogurt containing probiotics	5	4	3	2	1
I take a probiotic supplement	5	4	3	2	1
I use a traditional approach to cleaning my home- the way my mother and grandmother cleaned.	5	4	3	2	1
I limit or avoid the use of antibiotics	5	4	3	2	1
I own a cell phone and use it many times per day.	5	4	3	2	1

Food and environmental exposure

48. Have you been hospitalized for more than 24 hours in the previous 3 months?
    - Yes
    - No
49. Have you or anyone in your household had outpatient surgery in last 3 months?
    - Yes
    - No
50. Have you visited a patient in a hospital or long term care facility (such as a nursing home) in the past 3 months?
    - Yes
    - No

51. The purpose of this survey is to gather information that might relate to the bacteria that we find on your body. With this is mind, is there anything else that you can think of that we did not ask about that might cause the skin on your hands to be different than other people? (write in)

Proceed to END if under the age of 18

52. How often do you handle raw/uncooked pork products?
    - I do not handle pork products
    - Less than once per week
    - Approximately once per week
    - 2-3 times per week
    - more than 4 times per week

53. How often do you handle raw/uncooked chicken products?
    - I do not handle pork products
    - Less than once per week
    - Approximately once per week
    - 2-3 times per week
    - More than 4 times per week

54. How often do you handle raw/uncooked turkey products?

I do not handle pork products
Less than once per week
Approximately once per week
2-3 times per week
More than 4 times per week

55. Do you currently smoke cigarettes/ cigars?

Yes (how many per day?)___________________________
No

55a. Have you ever smoked cigarettes/ cigars?

Yes (year quit)______________
No

56. Do you currently use chew tobacco?

Yes
No

56a. Have you ever used chew tobacco?

Yes
No

57. Have you spent time in a jail or other correctional facility in the past 3 months (as visitor or inmate)?

Yes
No

58. Do you work or volunteer in a hospital, physician's office, or long-term care facility currently?

Yes
No

59.. In the past 3 months have you been in contact with any of the following types of live animals? (check yes or no for each)

Chickens	yes	no
Cattle	yes	no
Swine (pigs)	yes	no
Horses	yes	no
Goats	yes	no
Sheep	yes	no
Turkeys	yes	no
Cats	yes	no
Dogs	yes	no

Other type of animal, please specify _______________________

END. Thank you for completing this survey!
