# Supplementary material for: Staphylococcus aureus carriage is associated with microbiome composition in the nares and oropharynx, not the hand, of monozygotic twins
Source: Front Microbiomes. 2025 Jan 20;3:1457940. doi: 10.3389/frmbi.2024.1457940 (PMC12993631; doi:10.3389/frmbi.2024.1457940)
Supplement: Supplementary file 2 [file DataSheet2.pdf]

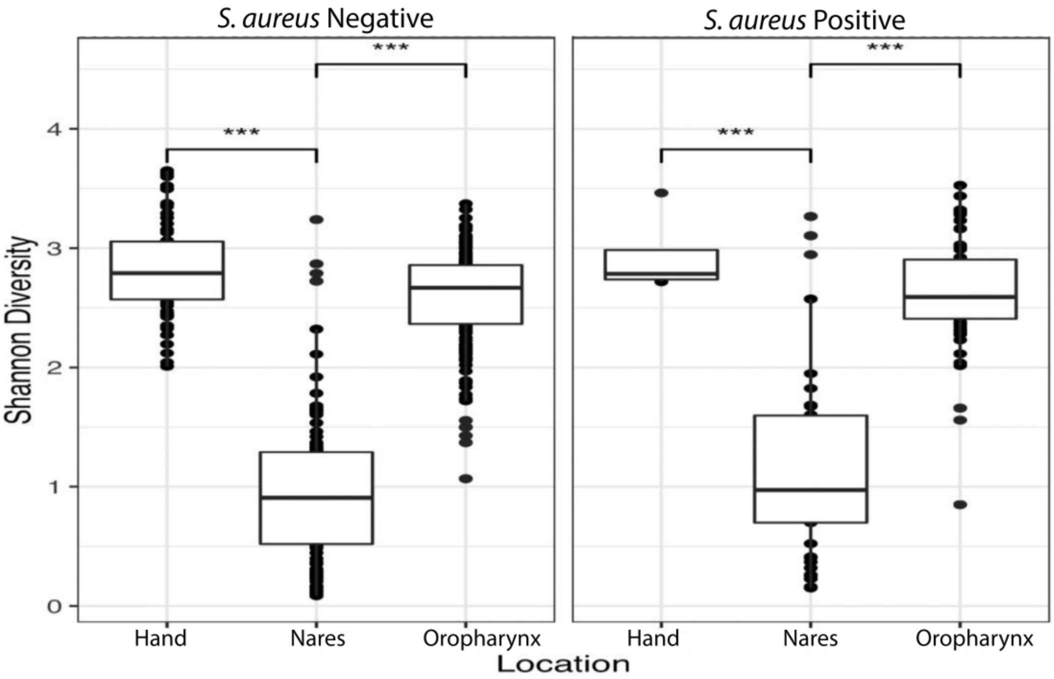
S. aureus Negative
S. aureus Positive
***
***
***
***
Shannon Diversity
0
1
2
3
4
Hand
Nares
Oropharynx
Hand
Nares
Oropharynx
Location

A

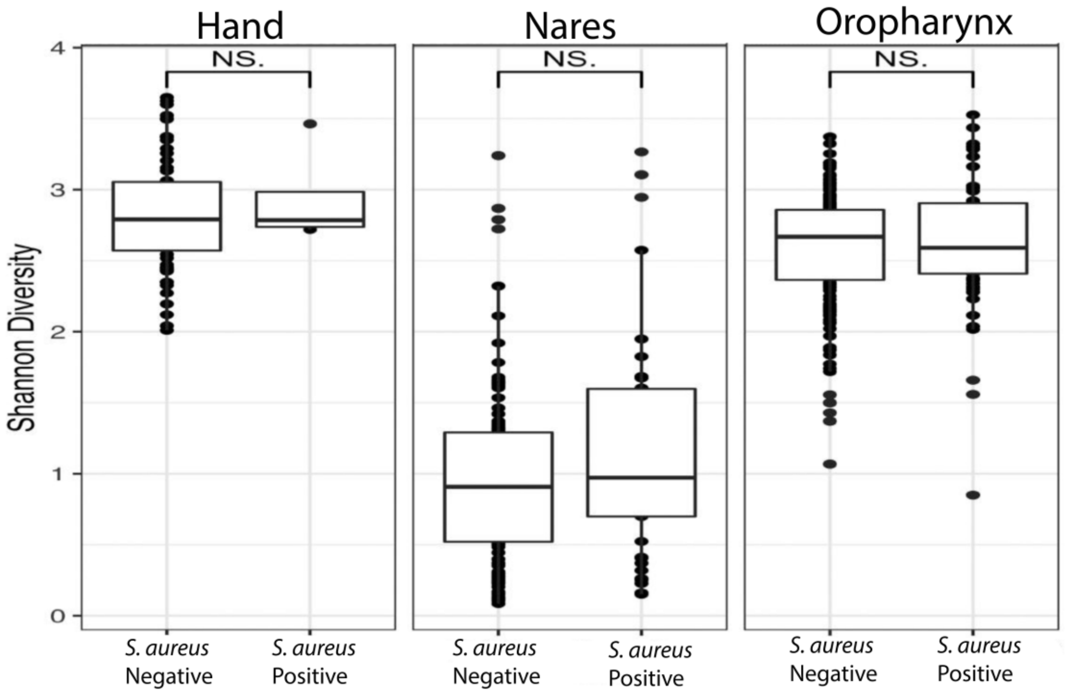
Hand
Nares
Oropharynx
NS.
NS.
NS.
Shannon Diversity
0
1
2
3
4
S. aureus Negative
S. aureus Positive
S. aureus Negative
S. aureus Positive
S. aureus Negative
S. aureus Positive

B

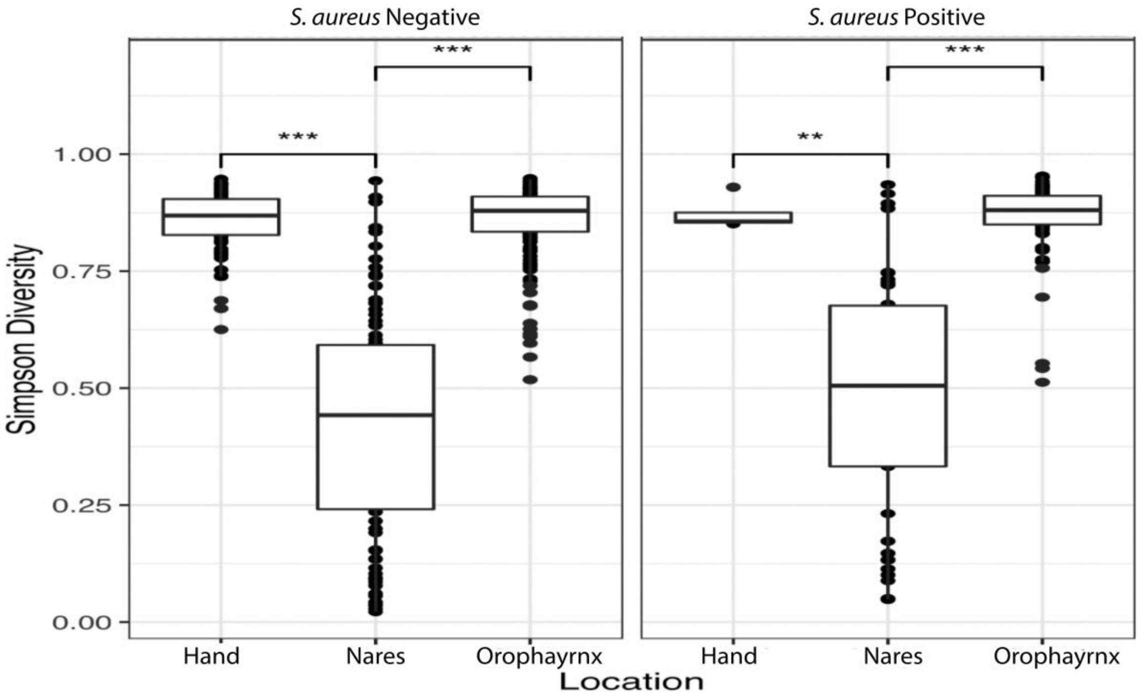

C

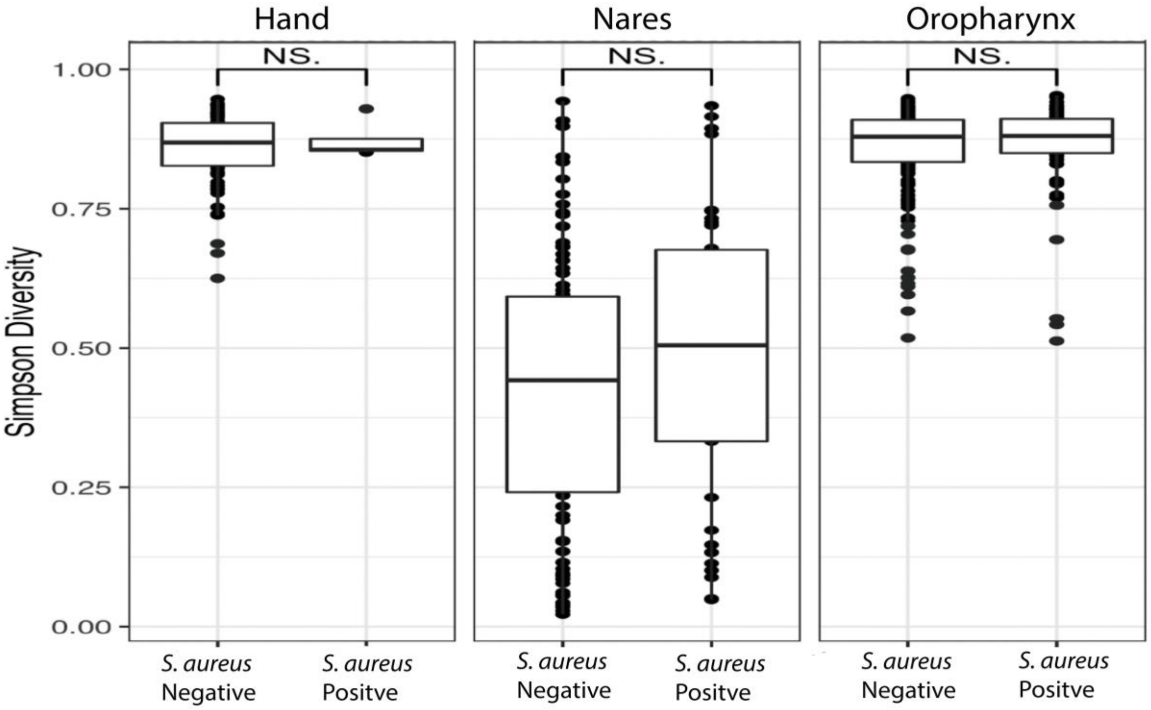

D

Supplemental Figure 2. Shannon and Simpson index analyses of Monozygotic twin participants' microbiome from the Nares, Oropharynx, and hand carriage separated based on *S. aureus* carriage status and by sample location (Shannon index A-B and Simpson Index C-D).
