# Supplementary material for: Staphylococcus aureus carriage is associated with microbiome composition in the nares and oropharynx, not the hand, of monozygotic twins
Source: Front Microbiomes. 2025 Jan 20;3:1457940. doi: 10.3389/frmbi.2024.1457940 (PMC12993631; doi:10.3389/frmbi.2024.1457940)
Supplement: Supplementary file 3 [file DataSheet3.pdf]

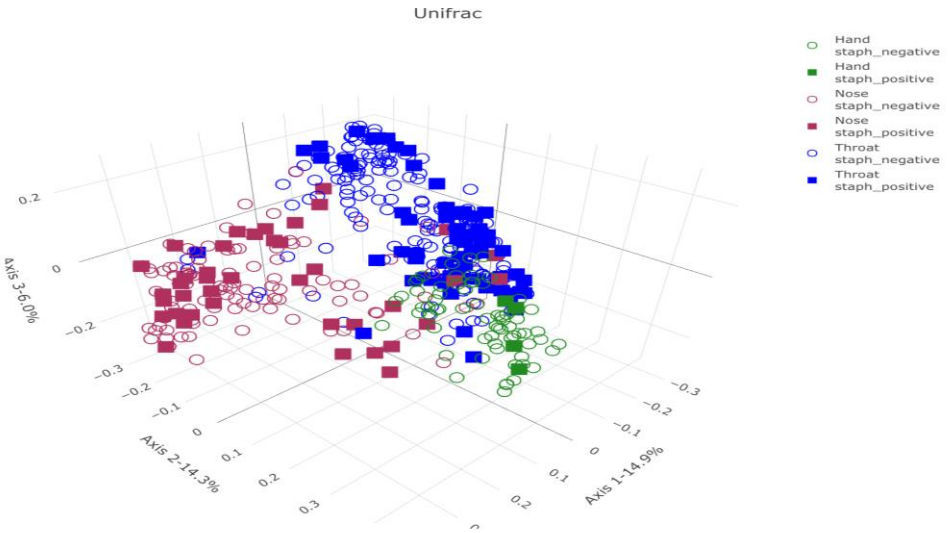

	Df	SumOfSqs	R2	F	Pr(>F)
Location	2	25.93214	0.212244	59.43051	0.001
Staph.culture	1	0.689261	0.005641	3.159255	0.001
Residual	438	95.5593	0.782115		
Total	441	122.1807	1		

A

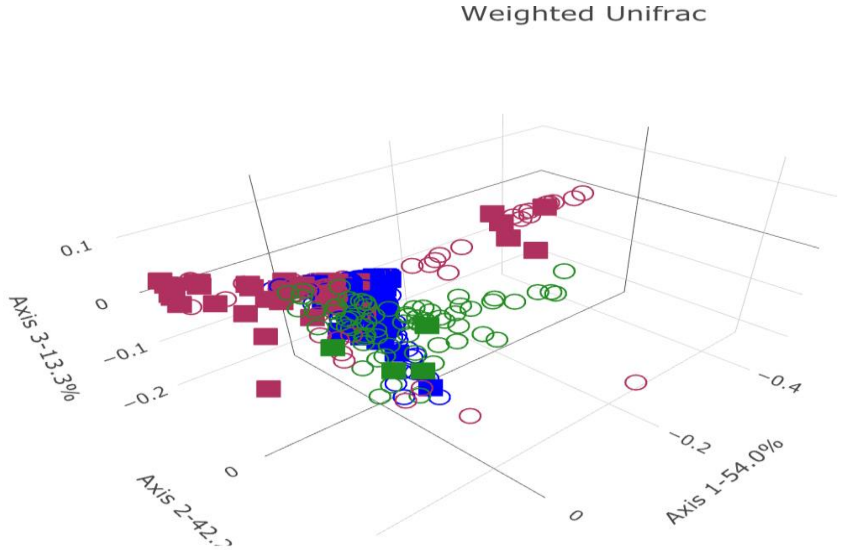

	Df	SumOfSqs	R2	F	Pr(>F)
Location	2	0.652862	0.141895	36.79452	0.001
Staph.culture	1	0.06235	0.013551	7.027993	0.003
Residual	438	3.88582	0.844554		
Total	441	4.601033	1		

B

Supplemental File 3- Figure 3. Principal Coordinates Analysis (PCoA) of Monozygotic twin participants by location and *Staphylococcus aureus* culture-dependent status: A) UNIFRAC and B) Weighted UNIFRAC and corresponding PERMANOVA with interaction.
